# IMPROVING FUTURE PLANNING AMONG OLDER ADULTS WITH VISION-THREATENING EYE DISEASE

**DOI:** 10.1093/geroni/igae098.4219

**Published:** 2024-12-31

**Authors:** Silvia Sörensen, Yunxiang Chen

**Affiliations:** University of Rochester, Warner School of Education and Human Development, Rochester, New York, United States; University of Rochester,, Warner School for Education and Human Development, Rochester, New York, United States

## Abstract

Preparation for Future Care Needs (PFCN) can protect older adults’ well-being as they face age-related challenges like Age-related Macular Degeneration (AMD), which progressively impacts the loss of their activities, functioning, and quality of life. However, rates of PFCN are lower among AMD patients than non-diagnosed community-dwelling older adults. We examined whether PFCN can be increased in a sample of 180 AMD patients aged 60-96 (63% women). Participants were randomly assigned to an 8-week in-home Preventive Problem-Solving Intervention (PREPSI), designed to enhance PFCN, or to an in-home Life Review intervention (attention control). Both conditions received 4 weeks of vision loss education before the intervention. We used Latent Growth Modeling to test whether the intervention would lead to change in the trajectories of the five dimensions of PFCN compared to the control condition over 5 measurement points (two post-intervention). Trajectories were significantly different for PREPSI versus control participants on Decision Making (DM). DM improved significantly for the intervention group and continued to improve at 6 month follow-up (p<.05). Although the trajectories for Concrete Planning (CP) diverged, they were not significantly different from one another (Wald χ2 =1.511, p=.219). Gathering Information (GI) improved significantly for both PREPSI and control group. Awareness showed significant and surprising decrease only in the intervention group, whereas Avoidance showed non-significant changes. Within the intervention group, covariate analyses showed that older participants had slower rates of GI increase and men had a faster increase in CP than women. PREPSI effectively increases engagement with future care needs for this population.

